# Sanitary

**Published:** 1873-09

**Authors:** 


### *Sanitary.*

The note of warning sounded by the Journal in the early part of the season, concerning the danger to public health threatened by the filthy condition of the city, has been obeyed too late by the Board of Health, which, with its usual tardiness, has attempted to shut the stable door after the steed has been stolen. It has commenced its work of "prevention" when the pestilence is in our midst.

The Board has, at last, after months of delay, appointed a Sanitary Superintendent who can legally perform the functions which have, for the past four months and a half, been subjected to maladministration in defiance of law.

The newly appointed officer, Dr. Ben. C. Miller, accepts the position under the most unfavorable auspices, with an epidemic of cholera threatening him in front, with "confusion worse confounded" in his rear; without practical experience in the duties of his new position, and with the odium of public distrust and contempt which has been earned for the position; he has before him an herculean task, viz., to reduce official chaos of six years accumulation to order, to lay the foundation for a system of sanitary statistics, to fight the pestilence, and to restore to public confidence that which has thus far merited only ridicule and contempt.

To accomplish all these, Dr. Miller brings into the contest youth, energy, a large share of administrative and executive ability, ambition, industry, and integrity of purpose, and, what is perhaps quite as important, the prestige of success in the important official positions which he has hitherto filled. We wish him success most heartily, although we do not see how the wish can be realized under the operation of such a stupid enactment as that to which the Board of Health owes its existence.

The only sensible reform movement in sanitary matters in this city will be that which, beginning by abrogating the law which created the present Board of Health, thereby legislating it out of existence, continuing by utilizing the talent and ability now contained in the Health Department, by excluding the useless lumber which now encumbers it, shall conclude by organizing a responsible Department (not an irresponsible Board) which shall be, what this has never been, a conservator of public health and vital statistics. H.

